# Transcultural Perspectives in Nursing: Understanding the Role of Healers and the Evil Eye in Modern Healthcare

**DOI:** 10.3390/nursrep14030181

**Published:** 2024-09-19

**Authors:** Felice Curcio, Hafsa El Khabir, Gianluca Chelo, Sonia Puggioni, Marica Soddu, Maria Raffaela Lucchetta, Cesar Iván Avilés-González

**Affiliations:** 1 Faculty of Medicine and Surgery, University of Sassari (UNISS), Viale San Pietro 43/B, 07100 Sassari, Italy; 2 University Hospital of Sassari, Viale San Pietro 10, 07100 Sassari, Italygian87.gc@gmail.com (G.C.); marica.soddu@aouss.it (M.S.); 3 Brotzu Hospital, 09047 Cagliari, Italy; sonia.puggioni@aob.it; 4 Department of Medicine and Surgery, Nursing Faculty, University of Cagliari, 09124 Cagliari, Italycesari.avilesg@unica.it (C.I.A.-G.)

**Keywords:** beliefs, evil eye, culture, health, qualitative research, traditional healers, transcultural nursing, nursing

## Abstract

Background: The belief in the evil eye is defined as the power to cause harm through ‘negative energy’ obtained through a glance. The practice of *“medicine of the evil eye or sa mexina de s’ogu”* by the *“feminas e ominis de mexina”* (healers) is so deeply rooted in the Sardinian culture that it influences health practices. Transcultural nursing, conceptualised by Madeleine Leininger, emphasises the importance of providing health care that takes into account patients’ cultural beliefs, practices and values. This study aims to explore, from the perspective of Madeleine Leininger’s transcultural nursing theory, the perception of the “feminas e ominis de mexina” practising “sa mexina de s’ogu”, in order to promote and raise awareness among health professionals of the importance of patients’ beliefs and cultures. Methods: A qualitative phenomenological study was conducted. Data were collected using semi-structured face-to-face interviews. The interviews were transcribed, read thoroughly, and analysed. Results: Fourteen healers were interviewed. Six main themes were extracted: (1) figure of the healer in its social context; (2) dynamics and methodology related to rituals; (3) effects of the Evil Eye; (4) ritual demand; (5) effectiveness of the ritual; and (6) comparison between the ancient and modern world. Conclusions: These findings suggest that healers act as central pillars in the connection between traditional medicine and religious practices. In response to the results obtained, it is essential to train healthcare personnel in transcultural nursing in order to provide care that is respectful and in harmony with the individual’s beliefs, promoting their comfort and well-being, and their health outcomes.

## 1. Introduction

The belief in the evil eye is defined as the power to cause harm through ‘negative energy’ obtained through a glance. It is a superstitious idea deeply rooted in many cultures around the world, including in the Sardinian population [1,2,3,4,5].

Regardless of scientific and historical advances, these beliefs persist and form an integral part of the culture of this Mediterranean island. On this island, the evil eye continues to be a living and influential practice, especially in rural areas [6]. The “*medicine of the evil eye or sa mexina de s’ogu*” is used to treat “*the evil eye or curse*”. It has millenary origins and is generally employed when Western or contemporary medicine does not provide answers to physical and emotional ailments such as headaches, insomnia, feelings of sadness and lack of luck [7]. Even today, the evil eye is a subject that is not discussed spontaneously in public, and many people consider it something stupid and ridiculous. However, in private and small-group settings, where trust and privacy are created for dialogue, many people discuss and debate the effectiveness of the phenomenon [8].

The practice of “*medicine of the evil eye or sa mexina de s’ogu*” is so deeply rooted in Sardinian culture that it influences its health practices. Often, when people suffer from some discomfort or illness, they turn directly to experts in this practice (healers) known as “*feminas e ominis de mexina*” (healers); only then, if the symptoms persist, do they turn to modern or Western medicine. This behaviour underlines the need for a holistic approach that recognises and respects the beliefs, rituals and cultural customs of individuals, in this case, patients [9]. The “*feminas e ominis de mexina*” are the central figures in the management of these disorders, offering specific rituals to eradicate the evils associated with the evil eye [10]. However, Western medicine decodes these symptoms associated with the evil eye to diagnoses of depression, anxiety, and stress (ethics). In contrast, traditional Sardinian medicine decodes it as the result of negative energies, curses or bad luck. These two ways of interpreting evidence highlight the need for a holistic approach that connects the elements of both conceptualisations to provide comprehensive and culturally sensitive care [11].

Madeline Leininger’s transcultural nursing theory emphasises the importance of nursing care incorporating the beliefs, practices and values of the patient’s culture [12]. Leininger’s model identifies transcultural care as fundamental to providing care that is respectful and in harmony with the individual’s beliefs, promoting their comfort and well-being, and their health outcomes [13]. Integrating local traditions into nursing care plans enables health professionals to provide culturally sensitive and more effective support. The application of the model promotes greater adherence to the care provided and improves the well-being of the individual as a patient [14].

Leininger’s concept of “Cultural Care” aims to identify, respect and value local traditional practices that are important to people. Recognising the influence these beliefs can have on health habits, behaviours and perceptions, the model integrates these practices into the planning and delivery of care [15]. This enables nurses and healthcare professionals to provide more effective and efficient care with greater sensitivity to cultural differences and in harmony with the needs of each individual as a patient [16]. In this way, transcultural nursing promotes health care that respects cultural differences, emphasises health promotion, and increases patient adherence to the treatment and care offered, where patients can recognise that the health care personnel respect and adopt their traditions [17].

It is estimated that in Sardinia, mainly in the centre of the island, many *“feminas e ominis de mexina”* still practise the “*medicine of the evil eye or sa mexina de s’ogu*” and therapeutic magic rituals are widespread. Moreover, many people believe that these therapeutic magic interventions produce beneficial results in certain physical and emotional disorders. Therefore, the aim of this qualitative research is to analyse and interpret, from the perspective of Madeleine Leininger’s transcultural nursing theory, the perception of the *“feminas e ominis de mexina”* practising “*sa mexina de s’ogu*”, in order to promote and raise awareness among health professionals and nurses of the importance of patients’ beliefs and cultures.

## 2. Materials and Methods

### 2.1. Design

This study was conducted using interpretative phenomenological analysis (I.P.A.), a qualitative research approach that values ‘a detailed experiential account of the person’s involvement in the context’ [18]. Interpretative phenomenological analysis enables an understanding of the meanings of communication through the narration of participants’ experiences from cultural, social and personal perspectives [19,20]. Interpretive phenomenological analysis develops a double hermeneutic circle; on the one hand, it adopts an idiographic approach because of the individual case investigation and, on the other hand, on the other hand, it uses an interpretive approach, following the principles of Husserlian hermeneutic phenomenology [21].

The study adhered to the Consolidated Criteria for Reporting Qualitative Research (COREQ) [22].

### 2.2. Sampling and Recruitment

A qualitative study was conducted in the province of Cagliari (Italy), in the towns of Sinnai, Settimo San Pietro, Selargius and Monserrato, from 1 July to 31 August 2023.

The researchers individually invited potential participants through targeted sampling. The participants were 14 *“feminas e ominis de mexina”* (healers), all of whom ranged in age from 25 to 75 years, and their educational level ranged from fifth grade to bachelor’s degree.

Healers eligible for the study had to meet the following inclusion criteria: (1) over 18 years old, (2) experienced in the practice of eye medicine, and (3) residing in small towns in the province of Cagliari (Sardinia, Italy).

The enrolment of participants lasted until data saturation was reached. None of the recruited persons refused to participate in the study.

### 2.3. Data Collection

Data were collected in the province of Cagliari at a location agreed upon with the participants. Interviews were conducted in the participants’ natural environment, using their native language (Italian), through two researchers.

Following the chosen methodology, each researcher involved in the study performed bracketing prior to data collection, writing down ideas, preconceptions and beliefs about the phenomenon under study [23,24]. By performing this “reflective technique” prior to data collection and analysis, researchers can be more careful to avoid introducing bias that could negatively affect the research.

Data were collected using semi-structured, face-to-face and individual in-depth interviews. Each interview lasted between 30 and 50 min. This type of interview was chosen because it is particularly informative, allowing the researcher to create a framework for the topics covered. The semi-structured interview guide provides a clear set of instructions for interviewers and, at the same time, can provide reliable and comparable qualitative data [18]. After reading the relevant literature, the researchers developed a 21-question semi-structured questionnaire (Appendix A). Probing and clarifying were asked during the interviews to ensure that rich data information was obtained [25].

The pilot interview was submitted to two “feminas e ominis de mexina” from Maracalagonis (Cagliari), who did not suggest changes to questions that were clear and concise. The interviews were structured to understand how the respondents talked about the phenomenon being investigated, allowing them to respond in natural language and provide immediate answers.

### 2.4. Data Analysis

The interviews were audio-recorded, replayed several times, and transcribed in full by assigning identification numbers. Each interview was independently and thoroughly read and re-read by two researchers, initially noting the descriptive, linguistic and conceptual elements that emerged from the text [26]. Next, emerging themes were identified, organised in a table for comparison, and finally grouped within superordinate themes. Consensual validation was performed between the two researchers, with no disagreements emerging.

### 2.5. Rigour

This study’s rigour was achieved by applying the criteria of reliability, confirmability, credibility and transferability recommended by Streubert & Carpenter [27]. Data were used and transcribed without comments and as direct quotes from the semi-structured interviews. The inclusion/exclusion criteria, participant characteristics, context, data collection, and analysis procedures were detailed [28]. To minimise the risk of confirmation bias, the quotes were shared among the researchers, and finally, the results were shared with the participants.

### 2.6. Ethical Considerations

This study was conducted in accordance with the Declaration of Helsinki [29], Italian privacy law (Decree No. 196/2003), and General Data Protection Regulation (GDPR-EU 2016/679). It was not approved by a registered ethics committee.

The interviews were authorised by the interviewed healers, who declared cooperation in the project on a completely voluntary basis. The possibility of discontinuing participation at any time was also indicated. Before the interviews began, the purpose of the study, confidentiality of the data, voluntariness of participation, guarantee of anonymity, and participants’ verbal consent for the interview and data recording were communicated. Each interview was assigned a sequential alphanumeric code, with no possibility of identifying the participants.

## 3. Results

The data analysis identified six main themes: (1) figure of the healer in its social context; (2) dynamics and methodology related to ritual; (3) effects of the Evil Eye; (4) ritual demand; (5) effectiveness of the ritual; and (6) comparison between the ancient and modern world.

### 3.1. Figure of the Healer in Its Social Context

It often happens that the figure of the healer is confused with that of the “sorcerer”, one who engages in occult practices for profit. In popular tradition, the *“feminas e ominis de mexina”* is the one who, through prayers, helps the person suffering from an illness; the dividing line between the two figures is often somewhat blurred and uncertain. N2’s testimony helps to understand what the perception of the ritual is within the Sardinian community: *“this practice is generally seen very well, as white magic. Certainly not black magic, as they are prayers! (…) Some people classify us as people who do strange things. However, for the most part people see me very fondly, but some unfortunately also think I do strange things. I am not a person who hides, I always hold my head high because I have never hurt anyone. When I could, I always did good, and I am recognised for it. Those who practice this medicine are not sorcerers, they get paid and do certain things with the intention of doing harm”.* N4 reports: *“Just as there are people who work in the service of God, there are others, in contrast, who engage in black magic practices. I myself have been a victim of these practices, having personal and direct experience with what is called ‘un male fattu’ (evil done)”.* The other *“feminas e ominis de mexina”* interviewed fully endorse this thought, as N1 reports: *“we do not make invocations to extraneous spirits, but prayers to the saints, to Our Lady, to God; and if it does no good, it certainly does no harm”.* N12 expresses his own point of view saying: *“as you have seen I made the sign of the cross and prayers”.* N3 adds: *“I always say, it doesn’t hurt because it’s a prayer! I’m not naming demons; I’m naming the Saints… I don’t do strange rituals like voodoo (…). It’s a medicine you do for the good of others”.*

Healers in this area state that kinship is neither an indispensable element for the transmission of the practice nor a specific age for learning it. Instead, it is necessary that the person teaching the ritual be older than the learner. As reported by N14: *“it was transmitted to me by a very old person who could no longer perform the ritual. (…) he proposed that I learn the ritual (…). I accepted very willingly because when it comes to doing good for others, one must never back down”.* The healer reports that she learned this practice as an adult because, as she says: *“you have to be aware of what you are doing*! N2 also learned medicine as an adult, but in this case the request came from her: *“it was a desire I had had for a long time. (…) the person I asked could teach me because I am younger”.*

N1 and N9, on the other hand, learned the prayers and use of ritual-related elements during their childhood. N1, retracing her memories, reports that she learned ritual-related prayer from her great-grandmother at the age of only eight or nine when she was still unable to fully comprehend its meaning. Moreover, it was her great-grandmother, being illiterate, who advised her to transcribe the prayer, which she would later teach and explain to her: *“write it down and I will tell you how to do it*”. Similarly, N9 also learned this practice at a very early age: *“when they taught it to me, I was seven years old. My grandmother taught it to me. (…) at the age of seven I was already performing the ritual”.*

The prayer that is handed down, as reported by N1, N6, N10 and N13, must first be read and, once learned, repeated in mind or in a whisper. This is to be jealously guarded in a place that no one knows. On the other hand, N2, N5 and N11 report: *“I was advised, once I learnt the prayer, to burn the paper it was written on! (…) I do not know why, perhaps so that it would not fall into the wrong hands. It must remain a secret”.* Finally, N4 and N8 report that for the completion of the ritual he must always read his own prayer: *“just as the priest in church reads the Gospel, which he knows by heart, I read my prayer in a low voice and must then keep it in a place where no one knows it”.*

The final point concerns the possibility of continuing to practise ‘sa mexina de s’ogu’ (evil eye) once ritual practice has been handed down. Several discordant opinions emerge from the interviews. N3, N6 and N12 report that the old lady who passed on this knowledge, once passed on, could no longer practise it: *“from the moment she taught it to me, she no longer practised it. If you teach it, you can no longer perform it”.* Other *“feminas e ominis de mexina”*, on the other hand, share the common thought that they can continue to perform the ritual even after passing it on.

### 3.2. Dynamics and Methodology Related to Ritual

In the territory of the province of Cagliari, the ritual of the evil eye, including the objects/materials used for its execution, is highly variable. According to almost all respondents, materials such as “water, coarse salt and grain” are needed to perform the ritual. In contrast, the rituals performed by N3 and N7 are different; they state that they do not use any items to bring the ritual to life: *“I do not use any items to perform the ritual, I just place my hand on the person’s forehead and that’s all. I realize the presence of the evil eye by the appearance of yawns. During the ritual I yawn repeatedly until my eyes water! (…) My medicine is different from the others”.*

The 14 *“feminas e ominis de mexina”* interviewed report that the ritual always begins with the “Sign of the Cross”, which will be re-performed on each item used to bring the ritual to completion and possibly, if physically present, also on the head of the person affected by the Evil Eye. This gesture implies a strong attachment to the Christian religion; in fact, according to N11, *“this practice is done as a function of God”.* The ritual consists of the interpretation of signs coming from the elements used; the healer, by interpreting these signs, is able to formulate a “diagnosis” and the consequent “therapy”.

In rituals using water, salt, and grain, water is poured into a glass, and inside it, “*4 grains of coarse salt and 8 grains of grain*” are introduced for N1, while for N2 *“4 and 9 grains of grain”* are dipped, respectively. The sign that indicates whether the person has been caught by eye, as reported by N6, N8, N9 and N12, is represented by the release of bubbles from the grain of wheat. Some add that the position taken by the grain of wheat inside the glass is very important: *“it reveals which position of the body has been affected by the evil eye”.* Moreover, as N13 reports, the shape the bubbles take makes it possible to reveal the gender of the person who cast the Evil Eye: *“small bubbles represent man, large bubbles represent woman”*. The ritual performed by N4, on the other hand, consists of the use of oil, water and salt, which are poured into a glass of water: *“(…) I take 4 grains of coarse salt and pour them inside the glass, making the Sign of the Cross, (…) I then dip my finger in the oil and drop 4 drops into the glass, causing them to fall so as to form a cross”.* The indicative sign that a person is under the influence of the evil eye is represented by the complete dissolution of oil inside the glass. According to N4, the ritual begins with the Sign of the Cross, which will then accompany each act of the ritual, followed by prayers, and recited in silence. The ritual continues until the drops of the oil form a cross, an indication of the effectiveness of the ritual and the removal of the evil eye.

An indispensable and fundamental element for healers to perform the ritual is knowing the name of the person affected by the evil eye. The ritual can take place even if the person affected by the evil eye is not present and does not physically participate in the ritual. The *“feminas e ominis de mexina”*, in this regard, show differing opinions as to how the ritual is performed at a distance. N5 and N14 require the name, surname and, if possible, a photograph of the person affected by the evil eye: “tell me your name and surname and I will perform the ritual (…) if I can have a photograph, it is better”. Other healers, on the other hand, do not need any photographs; moreover, it is enough for them to know only the person’s name, whether or not they personally know the person to whom the ritual is dedicated.

Analysis of the interviews reveals one point in common among all the healers: the reference to the number three, which can be traced back to the Trinity. N8 affirms that the ritual can be performed for a maximum of three consecutive times, after which she cannot continue: *“I have to stop, I cannot do it anymore, after the third one I have to stop. (…) Usually, to re-perform the ritual I wait 1–2 h, and to repeat it I change my clothes; it’s like wearing a new armor”.* N5 and N12, on the other hand, refer to the number three in relation to something else. Following the performance of the ritual and the formulation of the *“diagnosis of the evil eye”*, they keep the elements used—a glass of water with grain and salt—for three days, with the purpose of verifying the persistence or disappearance of the evil eye. In case the first ritual is not resolved, the healer will proceed with the performance of a new ritual. However, N3 has a different connection with the number three: *“I have to do three prayers, then another prayer in Sardinian repeated three times, which speaks of the saints who should eradicate the evil eye”.*

Although there are several rituals for the evil eye, they all share a strong connection with the Catholic religion. Numerous saints are invoked during the ritual, for whom healers have a strong feeling of devotion. Necessary also is a reference to Jesus Christ and the Virgin Mary. As reported by N4, *“Virgin Mary and Jesus must always be named and, through Jesus, the grace to remove the evil eye is asked for”.* N11 affirms that faith is an indispensable element for the ritual to take place: *“when people come to me, I ask if they are believers, not in relation to the evil eye, but in God! I can’t do it if they don’t believe in my God. (…) I would do it wholeheartedly, but if you don’t believe there is no point”.* This is shared by N1, who reports that prayer is the basis; not believing in the Christian religion does not allow one to believe in the whole ritual *“if I don’t believe in God, why should I believe in this ritual? (…) My grandmother also always told me: if they are not believers, don’t perform the evil eye*”. N2 and N3, in contrast to the other *“feminas e ominis de mexina”*, state that it is only necessary to have faith to learn the practice; for the person requesting the ritual, it is sufficient that they believe in this ancient practice, it is not necessary to practice the Catholic religion. N2 reports: *“I have done it many times to people who do not believe”.*

### 3.3. Effects of the Evil Eye

*“One thinks of the Evil Eye when a person has headaches, nausea, intestinal problems, insomnia. It presents itself as a general malaise, and not even medication can solve it (…) but it also leads to a succession of negative events that undermine the person’s experience”.* N1’s words, shared by other healers, help us understand the symptoms caused by the evil eye. *“S’ogu pigau”* (the evil eye) affects people, animals, plants and objects indiscriminately, but the subjects most affected are children. As reported by N8, children, unlike adults, do not have the same capacity to express their discomfort, and the sign that reveals the presence of the evil eye is the clustering of eyelashes: “*the eyelashes are clustered together, as if they were sticky, but without having ocular secretions*”. The symptomatology of the child suffering from the evil eye is characterised by prolonged crying, fever, restlessness, and, above all, a marked asthenia that renders him inexplicably inert. As reported by the N4 *“feminas e ominis de mexina”*: *“the child does not react, he starts to be listless, to feel drowsy, as if he had a loss of strength, and sometimes even a fever rises. After performing the ritual against the evil eye, incredibly, everything passes”*.

The same symptomatology of physical deconditioning can be observed in animals. N12 recounts*: “I had a beautiful cat, a British shorthair. A friend came to visit me and when she saw the cat she came up to me and said: how beautiful, I haven’t seen such beautiful ones in a long time”. After she left, the cat remained motionless, asleep, without strength”.* The same happens with plants, N3 reports: *“all it takes is for one person to have negative influences and they go dormant”.* Whether it is a human subject, a plant or an animal, the ‘victim’ experiences a strong state of unexplained malaise, which, according to popular belief, only goes into remission after the performance of the evil eye ritual. As N7 reports: “*Last week, a dog from Suelli was sick. He was taken to the vet and was diagnosed with nervous gastritis. I was called by his owners, who asked me, after taking the dog to the vet without any benefit, if I could perform the ritual. I am not a doctor, so I don’t know if it was nervous gastritis, but the dog was still sick. After performing the ritual, at 11 o’clock at night I informed the owners that I had performed it, and they replied telling me that the dog had nothing left”.*

These are, therefore, evils that can strike anyone, curable only through the ritual of eye medicine, and endowed with saving power seen as an antidote to evil.

### 3.4. Ritual Demand

Analysis of the interviews revealed that among the Sardinian people, beliefs and superstitions concerning the evil eye, and consequently its therapeutic practice, are still highly rooted, especially in small towns. As N3 explains: *“The ritual of the evil eye is practised by almost everyone, especially in the villages. It is no longer practised in towns […] because the belief has been lost, but above all the people who knew how to practise it have been lost. In the cities they do not pass on rituals, perhaps they believe they are superior to people who live in small villages […]. I understand that people with a high level of education are sceptical, but apparently there is also something outside their study and knowledge”.*

It emerges that rituals and beliefs related to superstition are prerogative of small villages, places far from the hectic pace of cities. However, despite the fact that this practice is not passed on in more industrialised contexts, since there is an absence of healers and a greater sceptical component, it is not uncommon for cultured people living in cities to request this ritual. N3 also reports: *“For example, an oncologist working at the Brotzu Hospital in Cagliari came with her husband, also an oncologist, to request the ritual. The husband, however, was very sceptical and did not want his wife to request the ritual. I came up to him and said, look doctor, I want you to understand that if I do the ritual to your wife, I am not hurting her; but if you get a drug wrong you are hurting people […]”.* From this, it is evident that discordant opinions towards the ritual coexist within cities.

It is also noted that this suggestive belief greatly conditions people’s lives, who assiduously request the ritual and use amulets in the hope of keeping this evil away. N10, on this topic, reports: *“amulets are often used for protection, for instance “su coccu”—an obsidian stone—and the green bracelet, often used on new-born babies. However, these amulets do not make you immune; moreover, they must be blessed”.* On the other hand, N4 states that it is not important to use amulets, *“the important thing is to have any blessed object and wear it […].* All the *“feminas e ominis de mexina”* interviewed report that theirs is a practice that is requested and performed *“even every day, by people of all ages*”. Examining this aspect, N9 reports that *“there are many people who say to me: please, perform the ritual, I’m not well! These do not immediately seek medical treatment, but in the first instance request the ritual, as if it were the grace that cures all ills”.* This testimony allows us to perceive how much this superstition is alive in the hearts of the Sardinian people and how active and in demand this practice is even today.

### 3.5. The Effectiveness of the Ritual

After analysing what the ritual consists of and the connotations, negative or positive, that it has acquired over the centuries, it is good to ask whether the people who require it really need it. An inherent condition of human beings is that they attribute the causes of their illness to external factors. It often happens that many people convinced they have the evil eye, turn to healers, who, after performing the ritual, realise that the problem detected is not related to negative energy but to some other problem. As N6 reports: *“people tend to believe that the headache is necessarily due to the evil eye; instead, it may simply be stress accumulated during the day, but knowing that they are performing the ritual they feel better. (…) I think there are people who take it for granted that every little problem can only be attributed to the evil eye, and these can only be solved through praying”.*

Is this really a healing ritual? How do we explain that people get better despite the absence of an evil eye? Undoubtedly, many of our ailments often have a very high psychic component. This leads one to think that such a ritual could produce an effect that would reassure all those who request it. There are strong links between what happens in our minds and what our bodies manifest. In this regard, N1 reports: *“Many times it seems that the ritual works, although it is evident that the person is not the victim of the evil eye. After performing the ritual, they are well again, this is because they believe in it blindly”.* N12, on the other hand, states: *“many people request the ritual as a preventive measure, they even request it every day. That way they are well. By doing it every day, I don’t detect the presence of the evil eye; I often don’t yawn*”. N7, who has been a healer for over 20 years, reports that she has some doubts about this: *“‘sometimes people come to me and say that they have been put under the eye; I perform the ritual, even if it is not necessary, and many times it works anyway. People tell me they are fine. Sometimes I ask myself: is it the person’s self-deception at that moment or was it really the evil eye? Although, I observe that the drop of oil does not dissolve, so it was not evil eye”.*

Analysing the medicine of the eye from a psychological-scientific point of view, it could be said that many people who request this type of treatment, in reality, manifest illnesses linked to a strong psychic component. In fact, examining the symptoms attributed to the evil eye, these are attributable to pathologies with a relevant psychosomatic component, such as gastrointestinal disorders, headaches, insomnia and anxiety. These symptoms seem to disappear thanks to the trust and expectations of the person in the ritual performed by the healer. The medicine of the eye, therefore, could represent a brain-body response that promotes health and well-being, i.e., a ritual that succeeds in affecting the psyche and, in some cases, the body of man.

### 3.6. Comparison between the Ancient and Modern World

All *“feminas e ominis de mexina”* share the opinion that they do not want to replace medical practitioners, as the cause of illness cannot always be traced back to the evil eye. However, discordant thoughts emerge in relation to when to seek medical advice and take medicine and when to ask the healer for help. The question that arises spontaneously is: in the Sardinian population, when suffering from any kind of evil, do people tend to consult the healer or the doctor first? Which of these two therapies is initially preferred?

The N4 *“feminas e ominis de mexina”*, reflecting on this question, reports: *“usually they first take the medicine, then they call me and tell me that despite taking the medicine, days later, the problem has not gone away. I believe that by taking the medicine of the eye, if you don’t get well, it hurts less. However, if you take medicine you don’t need, that does hurt. Wouldn’t it be better to do the eye medicine first? If you don’t solve the problem later, you take the medicine”.* The experienced healer, therefore, emphasises that performing such a practice does not harm the person requesting it, as it is simply prayer. On the contrary, taking the wrong medicine can cause side effects. Therefore, she suggests taking the medicine only under the condition that upon finding the absence of the evil eye, the ritual has had no effect, and therefore, the person is suffering from an evil that cannot be attributed to it. On the other hand, N1 objects to this statement, reporting: *“often before doing eye medicine I ask the person if the discomfort can be attributable to other causes. I prefer to investigate the cause of the malaise first […]. I myself, before asking my grandmother to give me the eye medicine, take tachipirina (paracetamol) and wait for the discomfort to end. If I am still sick after two days, I tell her to perform the ritual.*

Again, N1 reports: “I’m young and I’m a little bit restrained towards practice. *[…] it’s the modern context that leads me to think this way”.* The context and experiences of the healer, N1, are very different from those of older *“feminas e ominis de mexina”*. Through N7’s testimony, one can understand how the performance of this practice represents a considerable commitment for the person performing it*: “It’s a strange feeling that you get. It’s not like a simple headache […] It is demanding. In fact, many times I think of passing it on and no longer performing the ritual”.* This testimony shows that although there is a good intertwining of the ancient and modern worlds in Sardinia, without the prevalence of one over the other, problems arise in relation to the readiness to perform the ritual. Everyone is aware that the ‘time factor’ is very important nowadays; the social and economic context in which we are immersed makes it difficult for healers to perform the ritual.

N1 reports: *“my grandmother receives many people every day. Being older she is better known and has more fame than me. I, on the other hand, or those like me who are young, tend to protect myself, because I have my own private life, and I can’t spend all day doing eye medicine. I lead quite a hectic life and, rather than refusing to perform the ritual, I delegate my grandmother or make sure that it is not known around… my grandmother is literally harassed*”. The story told by N11 relates a further anecdote: *“I attended a conference on ancient medicines a few years ago and showed my ritual; a journalist literally chased me to interview me. I only agreed to the interview on condition that he wrote down exactly what I said, otherwise I would have reported him. After the publication of the interview in a newspaper, for a few months, there were people queuing in front of my house to ask for eye medicine. I could no longer eat or sleep. This also caused me shame; I did not know what people might think of me. Moreover, because I worked at the commune, my neighbour was my secretary. People would even go to this neighbour’s mother’s house when I was not at home; people even came from Milan, Turin, (…)”.* This testimony allows us to fully understand the reality that *“feminas e ominis de mexina”* live in the modern world.

## 4. Discussion

Analysis of the semi-structured interviews conducted revealed that the *“feminas e ominis de mexina”* six main themes: the figure of the healer in its social context, dynamics and methodology related to ritual, effects of the evil eye, ritual demand, effectiveness of the ritual, and comparison between the ancient and modern worlds. It also emerges that the practice against the Evil Eye is still in high demand in Sardinia. The community assigns *“feminas e ominis de mexina”* the role of spiritual guides, who act as a central pillar in bridging traditional medicine and religious practices. The results of our study coincide with those of other research conducted in different contexts, both Mediterranean and global, which have analysed the healing of the evil eye in rural contexts, where ambiguity between spirituality and physical health is quite common. One example is de Maya Sánchez and Garre’s study [30], an ethnographic investigation in the village of Cehegín, Spain; the authors identified rituals for curing the evil eye, which include practices of syncretism and distinct Christian elements such as holy water and the cross. This is similar to our qualitative study in Sardinia; however, unlike other cultures, *“feminas e ominis de mexina”* has strong roots in Catholic Christianity, creating a very idiosyncratic phenomenon.

Berger, in his article on the superstition of the evil eye, also emphasises how these practices can vary culturally, although in many places, they are connected to a mystical-religious tradition [31]. For this reason, Madeline Leininger’s theory of transcultural nursing provides an interpretation of this phenomenon, understanding the importance of respecting and accepting cultural practices as significant elements of the health of individuals and communities. This is confirmed by Pasquale’s study on the impact of the evil eye on community health nursing [32]. Therefore, nurses should be well aware of this reality and understand it through this nursing theory. Nurses should develop a broad dimension of health and illness that is not only based on Western medicine, but also by familiarising themselves with rituals and concepts of health and illness of mystical-religious origin, respecting the cultural concepts of individuals and communities. Touhami, in his article on the transfer of beliefs about the evil eye among Maghrebi youth in France, identifies how these concepts influence the understanding of health in immigrant communities, encouraging healthcare workers to understand cultural aspects in order to provide more empathetic and effective care [33]. This would promote higher levels of trust and integration with the people being cared for while encouraging adherence to modern medical treatments.

In health professionals, it is essential to promote transcultural nursing literacy for the practice of nursing care in both hospital and out-of-hospital settings, promoting skills to foster care for individuals, families and communities based on evidence-based nursing care. The creation of healthcare guidelines or protocols on common cultural beliefs in regions with geographical or cultural similarities promotes the empowerment of the nursing profession. It creates a harmonious relationship between nursing professionals and the individuals, families, and communities they care for, as shown in studies by Herrero-Hahn et al. [34] and Douglas et al. in their book on Global Applications of Culturally Competent Health Care: Guidelines for Practice [35]. Other strategies that can help transcultural nursing to be more effective include actions in which emphasis is placed on cultural anthropology and health in the education of future nurses. For example, contextualising transcultural nursing theory to the reality in which students find themselves; teaching how to interact respectfully, consistently relating in nursing and medicine based on scientific evidence, as highlighted by Langton’s 2018 analysis [36]; generate trust in patients, improving adherence to therapeutic treatments based on scientific evidence. In addition, it would be desirable to create educational materials [37] and simulated scenarios for virtual active learning [38], and to maximise the acquisition of knowledge and skills regarding transcultural nursing care in both hospital and out-of-hospital settings, as highlighted by the study by Douglas et al. [35]. With regard to health policies, on the other hand, cultural mediators already exist today; however, specific training on topics such as the evil eye, courses in cultural anthropology, and health would help to promote more effective work, as highlighted in the study by Venables et al. [39] and Peres & Sharaby [40].

The study conducted in Ghana by Bell et al. [41] highlights a deeply rooted cultural aspect within their society that directly influences health decisions, particularly when Western and traditional medical practices intersect. In light of this, it is important to consider the risk that excessive reinforcement of beliefs may lead to conflict with evidence-based medical interventions. This has been extensively documented in the literature. For example, in a study conducted in Turkey, patients with epilepsy with superstitious attitudes used magic to cure their illness instead of following medical instructions [42]. In the study by Taher et al. 40% of patients attributed their physical illness to superstitious thoughts such as the ‘evil eye’; furthermore, patients with hypertension who believed in superstition did not adhere correctly to their treatment regimen [43]. Abrehderi et al. found that diabetic patients with superstitious beliefs had poor self-care [44]. Finally, Omeje & Nebo showed that people with beliefs did not correctly follow medical instructions and prescribed medication [45].

Although this study provides a more detailed view of the practices of the *“feminas e ominis de mexina”,* the sample is limited to the province of Cagliari, Sardinia, Italy. It is essential to clarify that the island has eight provinces, and the study has only been conducted in the province of Cagliari, which limits the generalisation of the results to other contexts or provinces of the island and Italy. Further studies should be conducted in other provinces to identify the differences and similarities in this phenomenon. Additionally, it is crucial to investigate the practice of “*medicine of the evil eye or sa mexina de s’ogu*” from the perspective of those who seek this type of treatment or solution to physical health problems, emotional problems and spiritual well-being. It would be appropriate to compare interpretations across different demographic groups, as highlighted in the study by Mullick et al. [46], where aspects such as education and gender were identified as factors that influenced the perception and management of belief in the evil eye in the Bangladesh region. 

## 5. Conclusions

This research has shown the permanence of ancestral and traditional practices related to the evil eye on this Mediterranean island, emphasising their importance in the daily and cultural life of the region, regardless of the influence of contemporary life.

In nursing practice, it is essential to create literacy and education at the university level for future nurses and current professionals working in the healthcare system. Education that promotes a harmonious relationship based on cultural, anthropological and scientific principles in nursing, based on scientific evidence, so that nursing care is more effective regardless of existing cultural diversity. This work provides evidence that further confirms how Leininger’s theory provides an understanding of nursing from a transcultural perspective. It also promotes further investigation of the phenomenon in similar contexts, both in the Sardinian region and in the Mediterranean context, as well as internationally.

## Data Availability

The data presented in this study are available from the corresponding author upon request.

## Public Involvement Statement

The publication costs were funded by the Order of Nursing Professions of Sassari, Italy.

## Guidelines and Standards Statement

This manuscript was written following the recommendations of the COREQ guide for qualitative research reporting [22].

## Appendix A. Interview Guide

- What is the evil eye and what is it for?
- Origin of the practice.
- Methodology and materials used.
- How it is learned/transmitted.
- At what age can it be handed down/learned?
- What is the frequency of this practice (how many people perform it and how many request it)?
- How is it noticed that there is the presence of the evil eye?
- How is this practice viewed in one’s cultural context?
- How long does the ritual last?
- Are there rituals with preventive effect?
- Does the person to be healed have to be materially present?
- Can anyone become a healer? or are special gifts required?
- During the ritual, is reference made to the Christian religion?
- Is the evil eye practiced more by men or women?
- Can the evil eye be performed only on people or also animals and/or things?
- Are there subjects on whom the ritual cannot be performed?
- What are the symptoms of people affected by the evil eye?
- Does the rite require a monetary payment?
- Can the healer refuse to perform the rite?
- What is the average age of people who request the evil eye?
- Can people be asked to repeat the ritual?
